# Impact of p53 knockdown on protein dataset of HaCaT cells

**DOI:** 10.1016/j.dib.2022.108274

**Published:** 2022-05-15

**Authors:** Daniil D. Romashin, Alexander L. Rusanov, Peter M. Kozhin, Maxim N. Karagyaur, Olga V. Tikhonova, Victor G. Zgoda, Nataliya G. Luzgina

**Affiliations:** Institute of Biomedical Chemistry, 119121, Moscow, Russia

**Keywords:** Keratinocyte, p53, Proteome, LC-MS/MS, shRNA, Knockdown

## Abstract

The HaCaT line of immortalized non-tumor cells is a popular model of keratinocytes used for dermatological studies, in the practice of toxicological tests, and in the study of skin allergic reactions. These cells maintain a stable keratinocyte phenotype, do not require specific growth factors during cultivation, and respond to keratinocyte differentiation stimuli. HaCaT cells bear two mutant p53 alleles - R282Q and H179Y. At least two mechanisms of GOF (gain-of-function) of mutant p53 are known: it affects functions of p63/p73 by inhibiting their binding to DNA; or it binds to new DNA sites by interacting with other transcription factors (NF-Y, E2F1, NF-KB, VDR, p63). Proteins of the P53 family play an important role in the regulation of proliferation and differentiation processes of human keratinocytes. Proteomic study of HaCaT cells with *TP53* gene knockdown provides new data for understanding the limitations of HaCaT cells when using them as an experimental model of normal human keratinocytes.

In this article we present datasets obtained through the high-throughput shotgun proteomics analysis of human immortalized HaCaT keratinocytes and p53 knockdown HaCaT keratinocytes. As a protocol for proteomic profiling of cells, we used the approach of obtaining LC-MS/MS measurements followed by their processing with MaxQuant software (version 1.6.3.4). The “RAW” files were deposited to the ProteomeXchange with identifier PXD033538.

## Specifications Table

**Table:** 

Subject	Biology
Specific subject area	Biochemistry, omics analysis, Biotechnology
Type of data	Table Text
How the data were acquired	Liquid chromatography-tandem mass spectrometric analysis was carried out using Q Exactive high-resolution mass spectrometer (Thermo Fisher Scientific, USA) coupled with an Ultimate 30 0 0 Nano-flow HPLC system (Thermo Fisher Scientific, USA)
Data format	RAW
Description of data collection	- Cell cultivation. - Protein extraction. - LC-MS/MS analysis. - Data processing.
Data source location	V. N. Orekhovich Institute of Biomedical Chemistry, Moscow, Russia
Data accessibility	Data are available via ProteomeXchange with identifier PXD033538. https://www.ebi.ac.uk/pride/archive/projects/PXD033538 Raw Western Blot pictures and ELISA data are available in Mendeley repository http://dx.doi.org/10.17632/wjhtzjyy45.1

## Value of the Data

• Dataset represents proteomes of samples from human immortalized HaCaT keratinocytes and p53 knockdown HaCaT keratinocytes which can be compared to reveal differences between them.
• These data may be of value to the scientists involved in the development of skin models in vitro.
• These data may be of interest to the researchers interested in the processes of differentiation of keratinocytes.
• Protein profiles are available in the form of “RAW” data that can be further processed by researchers using their own bioinformatics algorithms and analyzed together with their own data.

## 1 Data Description

HaCaT keratinocytes are widely used in various research areas such as dermatology, toxicology, skin allergic reactions study [1]. HaCaT cells are considered as a reliable keratinocyte model, since they express stable phenotype and respond to the canonical keratinocyte differentiation stimuli [2]. However, HaCaT cells bear two gain-of-function (GOF) mutations (R282Q and H179Y) in TP53 gene, typical for spontaneously immortalized cell lines [3]. Several GOF mechanisms are described. Mutant p53 prevents p63/p73 binding with DNA [4]. Also, mutant p53 is capable to binding with new DNA sites through interactions with other transcription factors, including NF-Y, E2F1, NF-KB, VDR, p63 [5,6]. Proteomic studies of HaCaT cells with inactivated p53 may expand the understanding of limitations of HaCaT cell line as a keratinocyte model.

The dataset contains “RAW” and “*.txt” files obtained through the high-throughput shot-gun proteomics analysis of normal human keratinocytes and immortalized HaCaT keratinocytes.

Data is available via ProteomeXchange. Dataset covers 10 biological samples. Information about samples is presented in Table 1. The sample description is uploaded as a separate txt document ”sample_description.txt”.

**Table 1:** Data of cell samples.

Parameter	Wild Type HaCaT	p53 knockdown HaCaT
Number of samples	5	5
Number of technical repeats per sample	3	3-4

**Fig. 1:**
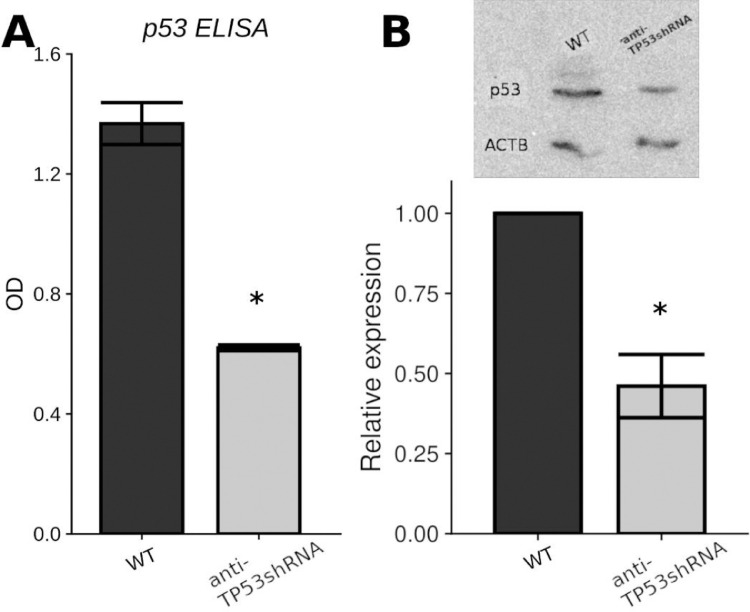
p53 expression in HaCaT cells after anti-TP53 shRNA lentiviral transduction. A) p53 ELISA B) Western blotting and densitometry results. WT – wild type HaCaT; anti-TP53 shRNA – p53 knockdown HaCaT. * Differences are significant relative to the WT group (p < 0.05).

ELISA and Western Blot were employed for knockdown verification. Transduction with anti-TP53 shRNA led to a significant decrease of intracellular p53 concentration (Fig. 1).

**Fig. 2:**
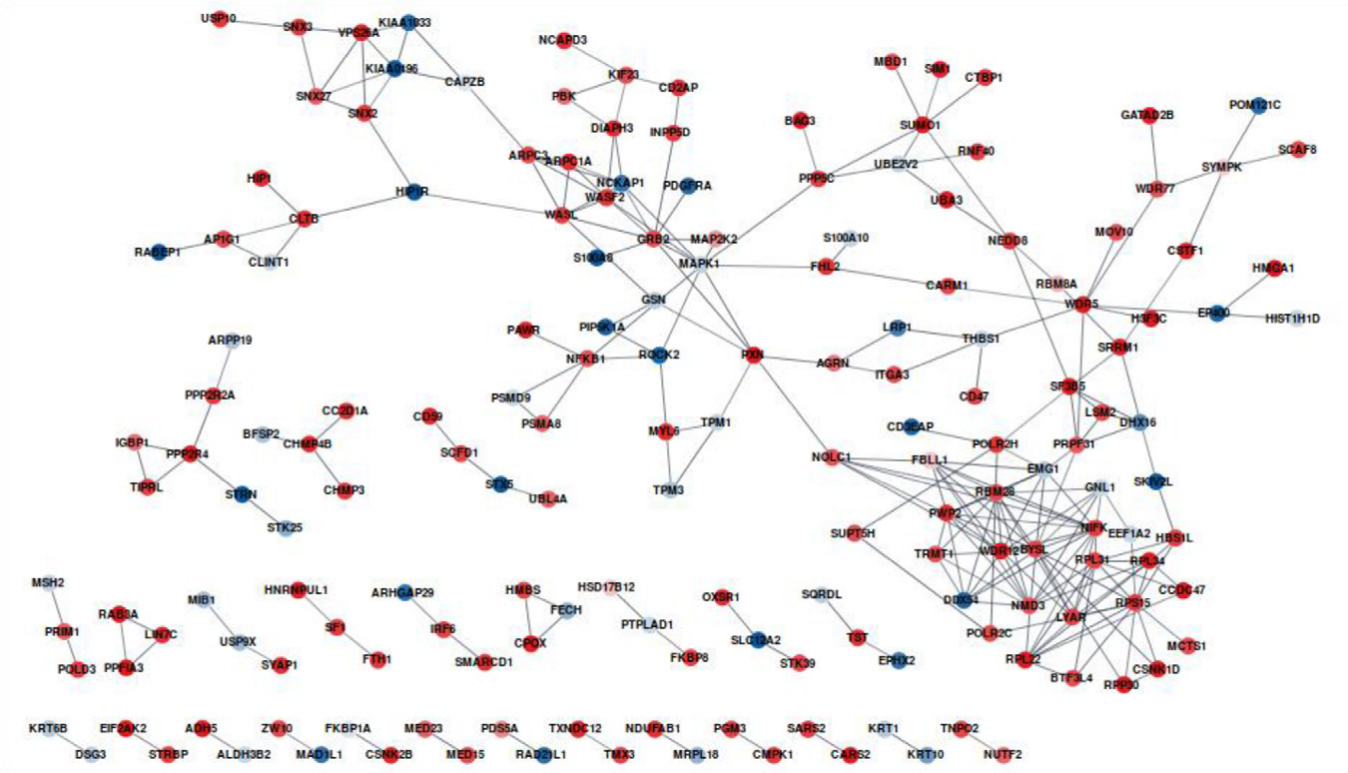
STRING network of p53 knockdown HaCaT proteins with significant regulation levels. Expression ratios were mapped to the nodes using blue-red gradient, where blue is related to the downregulated and red to the upregulated proteins. Proteins without interaction partners were omitted from the visualization. Network edges represent the confidence of interaction. The required interaction score was set to >0.7.

Comparison of wild-type and p53 knockdown HaCaT protein profiles resulted in the identification of 2875 proteins, out of which 320 were significantly changed, including 222 and 98 proteins that were up- and downregulated in p53 knockdown HaCaT, respectively. The STRING analysis of differently expressed proteins for p53 knockdown HaCaT is presented in Fig. 2.

## 2 Experimental Design, Materials and Methods

### 2.1 Reagents

Sodium chloride, taurocholic acid sodium salt (TCA), and acetonitrile were provided by Merck (Germany), formic acid was from ACROS Organics (USA), tris-(2-carboxyethyl)-phosphine (ТСЕР), methanol, trifluoroacetic acid (TFA) were from Fluka (Germany). Modified trypsin was obtained from Promega (USA). For cell cultivation, we used DMEM/F:12 medium (1:1), GlutaMAX, fetal bovine serum, PBS, penicillin/streptomycin (all from Gibco, USA).

### 2.2 Cells and culture conditions

HaCaT cells were obtained from Bank of DKFZ, Heidelberg and cultured in DMEM/F:12 medium (1:1, Gibco, USA) supplemented with 1% GlutaMAX (Thermo Fisher Scientific, USA), penicillin/streptomycin (100 UI/mL and 100 µg/mL, Gibco, USA) and 10% v/v fetal bovine serum (Gibco, USA). Cells were grown in 60 mm Petri dishes (Corning, USA) and harvested by trypsinization. The culture medium was replaced by fresh every other day.

### 2.3 shRNA gene knockdown

To knock down TP53 gene expression in HaCaT cell line lentiviral particles (LVPs) encoding anti-TP53 shRNA pLKO-p53-shRNA (Addgene, #25637) were used. To assemble the LVPs HEK293T cells were transfected with pLKO-p53-shRNA vector using the standard PEI transfection protocol [7]. The conditioned medium containing LVPs was collected 48-72 hours post-transfection and separated from cell debris by centrifugation at 4000g 4C° for 15 min. In order to increase the efficiency of transduction, protamine sulfate (50 ug/ml) was added to the medium containing LVPs. For transduction, we used HaCaT cultures with a confluent of 40-50%. The HaCaT medium was replaced with a medium containing LVPs, and the HaCaT plates were centrifuged for 1.5 hours at 800g RT. After centrifugation, the medium was replaced with a fresh HaCaT medium. Cells were incubated for 4 days at standard conditions with a regular medium exchange. To select the transducted cells, they were cultivated in a puromycin-containing medium (1 ug/ml) until all HaCaT cells in the control dish (transducted with lego-ig2 (Addgene, #27341 LVPs. This vector doesn't contain the puromycin resistance gene) were dead (approx. 10 days). Obtained cells were used for further experiments.

### 2.4 ELISA

For semi-quantitative determination of p53 protein level, Abcam ELISA kit (ab205713) was used. Cells were seeded into a microtiter plate at a density of 1 × 10^4^ cells per well 48 hours prior to analysis. Total cellular protein level was measured with a spectrophotometer. Samples were diluted with PBS to reach protein concentration of 200 µg/ml. Signal intensity was measured at 450 nm. Two biological replicates per group were used for ELISA. Difference between the groups was tested using Student's t-test. Difference was considered significant at p < 0.05.

### 2.5 Western Blot

Verification of knockdown was done using 8% sodium SDS-PAGE. 1 × 10^6^ of wild type and p53 knockdown cells were harvested by trypsinization for Western blot analysis. The cell pellet was lysed in RIPA buffer (150 mM NaCl, 1% Triton X-100, 0.5% sodium deoxycholate, 0.1% SDS, 50 mM Tris, pH 8.0).

The proteins were transferred onto the PVDF membrane following immunoblotting with anti-p53 (clone BP-53-12, PrimeBioMed, Russia) and β-actin (Bio-rad, VMA00048) antibodies as a loading control. Alkaline phosphatase-conjugated secondary antibodies (Bio-rad, STAR108A) were used for primary antibodies recognition. The chemiluminescent reaction was detected with DNR LumiBis Gel Imaging System 3.2. The images were obtained using GelCapture software. Three biological replicates per group were used for Western Blot. Difference between the groups was tested using Student's t-test. Difference was considered significant at p < 0.05.

### 2.6 Protein extraction for MS analysis

After reaching confluency of 75%, cells were washed three times with PBS and then harvested mechanically with cell scrapers (Corning, USA), pelleted with centrifugation, and incubated in 100 µL lysis buffer (4% SDS in PBS, pH 7.4) on an orbital shaker at room temperature for 20 min followed by a 5 min incubation at 95 °C.

Cooled down samples were sonicated three times at room temperature using Bandelin 2070 (Bandelin, Germany) for 20 seconds at 90% power.

Proteins were precipitated using the methanol-chloroform method [8]. The following sequence of actions was performed: 400 µl of methanol was added to 100 µl of each sample, the mixture was shaken on a vortex, 100 µl of chloroform, 300 µl of ddH_2_O were added, shaken again on a vortex, and centrifuged at 14000g for 2 minutes. Next, the upper layer (aqueous) was removed, and methanol was added in a volume of 400 µl. The resulting solution was vortexed and centrifuged for 5 min at 14,000 g to precipitate proteins. Methanol was carefully removed, and the precipitate was dried in an Eppendorf Concentrator 5301 (Eppendorf, Germany) at 45 °C for 5 min.

### 2.7 Sample preparation for MS analysis

Pierce™ BCA protein assay kit (Thermo Scientific™, USA) was used to determine the protein concentration. For this purpose, 20 µl of a denaturation buffer of the composition 5M urea, 1% TCA, 15% acetonitrile, 50 mM phosphate buffer, pH 6.3, 300 mM sodium chloride was added to 50 µg of protein extracts.

Further, to recover the protein, a 25 mM TCEP solution in 0.1 M ammonium bicarbonate was added in a volume of 5 µl for each sample and incubated at room temperature for 45 min. After that, a solution of 300 mm IAA in 0.1 M ammonium bicarbonate was added in a volume of 5 µl, incubated for 30 minutes in the dark, and the remaining IAA was suppressed with 5 µl of 300 mM DTT solution in 0.1 M ammonium bicarbonate. The volume of the reaction mixture was adjusted to 200 µl with 0.1 M ammonium bicarbonate solution, and trypsin was added in the ratio enzyme:protein 1:50.

Samples were incubated overnight at 37 °C with rotation. Samples were centrifuged at 10 °C and 12,000 × g for 10 min, then the supernatants were collected and purified using a C18 ZipTip according to the manufacturer's protocol [9]. Briefly, the columns were washed with 0.1% trifluoroacetic acid (TFA) in acetonitrile and equilibrated twice with 0.1% TFA in ddH_2_O, then the samples were loaded onto Zip-Tips and washed three times with 0.1% TFA and 5% methanol in ddH_2_O. The peptides were eluted with 70% acetonitrile with 0.1% formic acid. The elution of the peptides from the column was carried out with a solution of 0.1% formic acid in 70% acetonitrile.

### 2.8 LC-MS/MS analyses

Separation of the peptides was carried out on an Acclaim µ pre-column (0.5 mm × 3 mm, particle size 5 µm, inner diameter 75 µm; Thermo Scientific, USA). For this, a peptide solution (1 µg of peptide in 1-4 µl of 0.1% formic acid) was applied directly to the column. The concentration of peptides was performed at a flow rate of 10 µl/min in the isocratic mode of mobile phase C (2% acetonitrile, 0.1% formic acid) for 4 min. To separate the peptides, a high-performance liquid chromatography system Ultimate 3000 Nano LC (Thermo Scientific, Rockwell, IL, USA) was used. Separation was carried out on a C18 column (length 15 cm, inner diameter 75 µm, Acclaim® PepMapTM RSLC, Thermo Fisher Scientific, USA). Elution was performed at a flow rate of 0.3 µl/min with a gradient of 0.1% formic acid (buffer A) and 80% acetonitrile, 0.1% formic acid (buffer B).

Initially, the columns were equilibrated with buffer A for 12 min, and then, gradually (over 95 minutes), the concentration of buffer B was increased from 5 to 35%. Then for 6 minutes, the concentration of buffer B was increased to 99%. The columns were washed several times with 99% buffer B and equilibrated with buffer A for a7 minutes.

Mass spectrometer Q Exactive HF-X Hybrid Quadrupole-Orbitrap^TM^ (Thermo Fisher Scientific, USA) was used for MS analysis. Each sample was examined in three technical repeats. Analysis conditions: capillary temperature 240 °C, ionizing voltage 2.1 kV. Spectra were obtained in the range of 300-1500 m/z, resolution 120000 (MS). Tandem spectra were obtained in the range 140-2000 m/z, resolution 15000 (MS/MS). The maximum integration for precursor and fragment ions was 50 ms and 110 ms, respectively. AGC target was set to 1 × 10^6^ and 2 × 10^5^ for precursor and fragment ions, respectively. For the selection of the precursor, an isolation intensity threshold of 50,000 counts was determined. For fragmentation with high energy collisional dissociation at 29 NCE, up to 20 best precursors were selected. Precursors with a +1 and more than +5 charge state were excluded, and all measured precursors were dynamically rejected from triggering of a following MS/MS for 20 s.

The equipment of the “Human Proteome” Core Facility of the Institute of Biomedical Chemistry (Russia, Moscow) was used for mass spectrometric measurements. Data from proteomic studies were submitted to the ProteomeXchange Consortium through the PRIDE [10] partner repository (PXD033538 dataset identifier).

### 2.9 Protein identification

MaxQuant software (version 1.6.3.4) was used for MS/MS spectra identification. Proteins were identified using Homo sapiens reference proteome UP000005640, contained 20 361 reviewed and 58 691 unreviewed entries. The database was downloaded in July, 2021.

Following identification parameters were used for the database search: enzyme specificity - Trypsin, maximum cleavages allowed – 2, MS1 and MS2 tolerances – 5.0 ppm and 20 ppm, respectively, fixed modification - carbamidomethylation (Cys), variable modifications - N-terminal proteins acetylation and methionine oxidation (Met), false discovery rate estimated using the decoy hit distribution for Peptide Spectrum Matches (PSMs), peptides and proteins identification −1.0%.

## CRediT Author Statement

**Daniil D. Romashin:** methodology, investigation, resources, data curation, writing—original draft preparation, writing—review and editing; **Alexander L. Rusanov:** Conceptualization, methodology, investigation, resources, validation, writing—original draft preparation, writing—review and editing, project administration; **Peter M. Kozhin:** methodology, investigation, writing—original draft preparation, writing—review and editing; **Maxim N. Karagyaur:** methodology, investigation, writing—original draft preparation, writing—review and editing; **Olga V. Tikhonova:** investigation, writing—original draft preparation, writing—review and editing; **Victor G. Zgoda:** methodology, investigation, data curation, writing—original draft preparation, writing—review and editing; **Nataliya G. Luzgina:** Conceptualization, investigation, writing—original draft preparation, writing—review and editing, supervision, project administration.

## Ethics Statement

This work did not involve the use of human subjects or animal experiments.

## Declaration of Competing Interest

The authors declare that they have no known competing financial interests or personal relationships that could have appeared to influence the work reported in this paper.

## Data Availability

Protein dataset of immortalized HaCaT cells p53 knock down HaCaT keratinocytes (Original data) (ProteomeXchange).
